# Mucormycosis of Mandible with Unfavorable Outcome

**DOI:** 10.1155/2012/257940

**Published:** 2012-06-20

**Authors:** Nitin Prakash Oswal, Pushkar Kiran Gadre, Prachee Sathe, Kiran Shrikrishna Gadre

**Affiliations:** ^1^Nitin Prakash Oswal, Sahyadri Hospital, Bopodi, Pune 411003, India; ^2^Department of Oral and Maxillofacial Surgery, Sharad Pawar Dental College and Hospital, Sawangi Meghe, Maharashtra, Wardha, India; ^3^Critical Care, Ruby Hall Clinic, Pune 411001, India; ^4^Department of Oral and Maxillofacial Surgery, Bharati Vidyapeeth University, Dental College and Hospital, Katraj, Pune, India

## Abstract

Mucormycosis is a fulminant fungal infection that occurs most often in diabetic and immunocompromised individuals. Our patient, with uncontrolled diabetes mellitus and multiple systemic disorders, developed postextraction mucormycosis of mandible, an extremely rare complication. An initial clinical and radiographic diagnosis of mandibular osteomyelitis was made and the lesion was treated medically and surgically with curettage and saucerisation. The specimen was sent for histopathological evaluation, which showed necrotic area containing broad aseptate fungal hyphae with right angle branching consistent with mucormycosis. The patient succumbed to multipleorgan failure secondary to septicemia. The disease is usually fatal with a poor survival rate; there is still paucity of literature on the definitive management of this disease involving the mandible. This paper emphasizes the need for correction of underlying immunodeficiency and early diagnosis with aggressive multimodality treatment approach to offer the best chance of survival.

## 1. Introduction

Systemic fungal infections are almost always associated with morbidity and mortality. Amongst the opportunistic fungal infections, mucormycosis is the most tissue-destructive and life-threatening infection. Mucormycosis, also referred as zygomycosis/phycomycosis, was first described by Paultauf in 1885 [1]. Mucormycosis is mostly reported in patients with some form of immunosuppression, most commonly being uncontrolled diabetes mellitus or some type of debilitating disease, rarely a normal individual will ever present with mucormycosis. In the compromised host, mucormycosis results from altered immunity in which rapid proliferation and invasion of Mucorales organisms ensues in deeper tissues [2]. These fungi usually invade the body via inhalation, damaged or lacerated skin can also be a port of entry. The fungal hyphae invade the endothelium, producing thrombosis and infarctions resulting in gradual tissue ischaemia and necrosis of the affected structures.

The six accepted clinical types of mucormycosis are as the following.

1. Rhinocerebral mucormycosis, often associated with diabetes mellitus.
2. Gastrointestinal mucormycosis, associated with malnutrition, uremia, or Kwashiorkor disease.
3. Pulmonary and disseminated mucormycosis, associated with hematological malignancy.
4. Burn wound mucormycosis.
5. CNS mucormycosis.
6. Endocarditis and vascular mucormycosis following cardiac surgery [3].

Mortality is high, death can occur within several days to a few weeks, in spite of appropriate treatment being administered. The first recorded case of mucormycosis, which was in the lungs of a blue jay, was reported by Eisenberg et al. [3]. Rhinocerebral mucormycosis has been discussed comprehensively in the literature [4, 5].

But mucormycosis of the mandible is a rare entity [2, 6]. Following is a rare case of mucormycosis involving the mandible.

## 2. Report of a Case

**Figure 1:**
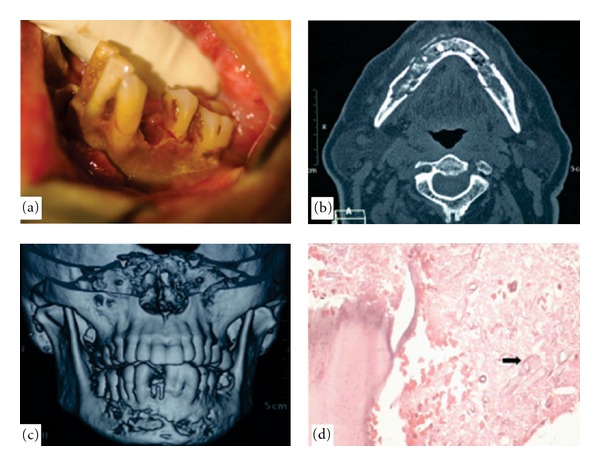
Showing clinical, radiological, and histological features of mandibular osteomyelitis due to mucormycosis. (a) Denuded avascular bone 36 to 32; (b) and (c) CT scan showing osteolysis of buccal and lingual cortices, loss of trabecular bone pattern, evidence of sequestra, and extent of mandibular involvement crossing midline; (d) photomicrograph showing broad fungal hyphae (black arrow) (Hematoxylin-Eosin stain, original magnification ×200).

A 68-year-old lady presented herself to our office with complaints of pain and foul smelling discharge from a nonhealing socket on the left side of posterior region of the mandible since 1.5 months. She had a history of surgical removal of left third molar (38) 2 months prior to her current visit. She had a significant long-standing history of diabetes mellitus, hypertension, ischaemic heart disease, diabetic nephropathy, as well as sleep apnea syndrome. According to the patient, she was on insulin therapy for the past 10 years and her diabetes was well controlled. Present clinical examination revealed the patient to be alert, oriented, febrile (100 F), and in severe pain on left side of face with parasthesia of lower left lip. Left submandibular nodes were palpable and tender. Intraoral examination revealed avascular denuded necrotic bone extending from 32 to 38 region. She was advised admission. On admission, insulin therapy was initiated in consultation with the diabetologist along with her usual medication (Nicorandil, Verapamil). Her laboratory investigation reports were as follows: total leukocyte count 14,300/cmm, hemoglobin 11.5 g/dL, platelet count 282,000/cmm, INR 1.1, sodium 140 mmol/L, potassium 3.9 mmol/L, random blood glucose 205 mg/dL, blood urea 73 mg/dL, serum creatinine 2 mg/dL, HbA1c 6.1%. Cardiac color Doppler revealed moderate pulmonary hypertension (RVSP-64 mmHg). Normal left ventricle size and left ventricular ejection fraction of 60%. Mitral valve prolapse with moderate mitral regurgitation. CT scan of mandible revealed an osteolytic lesion involving buccal and lingual cortices, loss of trabecular pattern of medullary bone, and multiple small air loculi with evidence of involucrum and sequestrum formation extending from left angle crossing midline and involving the body on the right side (Figure 1). 

 Considering her systemic condition and radiographic features a diagnosis of acute exacerbation of chronic osteomyelitis of mandible was considered. In joint consultation with the diabetologist, intensivist, anaesthesiologist, surgery was planned. Considering the extent of involvement of the mandible and the patient's apprehensions, it was decided to do broad surgical debridement of mandible to remove infected and devitalized tissue under general anesthesia. Insulin drip and empirical antimicrobial prophylactic treatment (with Piperacillin + Tazobactam 4.5 gm 8 hourly) was started. 

Curettage and saucerization of involved segment of mandible and total extraction of all teeth was done on the 2nd day of admission under general anaesthesia with nasotracheal intubation. Intraoperative findings revealed extensive necrosis of cortex and medullary bone and greenish discoloration of medullary portion of mandible suggestive of pseudomonas infection with multiple loose teeth. Swab from surgical site was sent for gram, acid-fast, and fungal staining with culture and sensitivity test. Necrotic tissue from the mandible was sent for histopathological examination. Patient was shifted to intensive care unit with endotracheal tube in situ. Postoperative course was uneventful for the rest of day. Extubation was done on the following day. Post-op days 2 to 4 were uneventful though she continued to have intermittent fever. Her blood and urine cultures sent on admission did not grow any organisms. Gram, Ziehl-Neelsen, and fungal staining of swab from surgical site revealed presence of few pus cells, plenty of gram-negative bacilli and few gram-positive cocci in pairs with no evidence of acid-fast bacilli or fungal elements. Culture from the swab grew multidrug resistant organism *Morganella morganii* ssp. *morganii*. Antimicrobial therapy was changed to Meropenem 500 mg 12 hourly and Teicoplanin 50 mg 24 hourly according to susceptibility report. She continued to have intermittent fever (99–101 F).

On postoperative day 5, she suffered from altered sensorium, decreased urine output and hypotension. She also had a spike of fever. Arterial blood gas revealed metabolic acidosis. She was intubated and put on inotropic support. Her laboratory reports showed WBC count 27,230/cmm, hemoglobin 9.4 g/dL, platelet count 305000/cmm, INR 2.14, derange renal and liver function tests (Blood urea 81 mg/dL, serum creatinine 3.9 mg/dL, total bilirubin 2.17, Alanine transaminase 626 U/L, aspartate transaminase 1243 U/L.) Diagnosis of septic shock with multiorgan failure (renal, liver and circulatory failure) was made and treatment initiated accordingly. Despite all efforts, she continued to deteriorate. On post-op day 7, histopathology report of the specimen of the mandibular bone showed presence of osteonecrosis with hemorrhage and necrotic area containing broad aseptate fungal hyphae with right angle branching consistent with mucormycosis. Lyophilized Amphotericin B was immediately added 50 mg 24 hourly. She suffered cardiac arrest on day 8 post-op and succumbed to septicemia with multiorgan failure.

## 3. Discussion

Fungi have been recognized as infectious agents for humans earlier than bacteria [7]. Mucormycosis incorporates a variety of infections caused by *zygomycetes*; a class of fungi that produce branching ribbon-like hyphae and reproduce sexually by formation of zygospores. Pathogen can be found in fruits, soil, feces, and can also be cultured from the oral cavity, nasal passages, throat of healthy disease-free individuals. *Mucorales *is a subtype of *zygomycetes*, which produces a *discrete* pattern of clinical infection. *Mucorales *is angiotropic, causing tissue necrosis, and are associated with disseminated and often fatal infections, especially in immunocompromised hosts. The fungi are normally avirulent, they become pathogenic only when the host resistance is exceptionally low. Ulceration in the mucosa or an extraction wound in the mouth can be a port of entry for mucormycosis in the maxillofacial region, particularly when the host is immunocompromised. Invasive mucormycosis is characterized by the rapid development of tissue necrosis resulting from incursion of blood vessels, ensuing thrombosis. Diabetes mellitus alters the normal immunological response of body to any infection in several ways. Hyperglycemia stimulates fungal proliferation, and the diabetic reduction in chemotaxis and phagocytic efficiency permit these otherwise innocuous organisms to thrive in acid-rich environment. In the diabetic ketoacidotic patient there is a high incidence of mucormycosis caused by *Rhizopus oryzae *because they produce the enzyme ketoreductase, which allows them to utilize the patient's ketone bodies [8]. It has been established that diabetic ketoacidosis momentarily disrupts the ability of transferrin to bind iron and this alteration eliminates a significant host defense mechanism and permits the growth of *Rhizopus oryzae *[9]. Successful management of mucormycosis largely depends on early diagnosis, reversal of underlying predisposing factors, prompt and ideally broad surgical debridement of infected tissue and rapid administration of systemic antifungal therapy. There have been no established regimes for the primary treatment of mucormycosis.

Most of the information of the efficacy of existing antifungal agents comes from a small series of cases, anecdotal case reports and in vivo studies of animal models of mucormycosis. Antifungal drugs have poor penetration ability at the site of infection. Surgical debridement of the infected tissue should be based on an emergency basis.

Removal of as much of the infected or devitalized tissue as possible while the infection is confined has the greatest benefit. The optimal therapy is ambiguous. The suggested antifungal therapy for mucormycosis is Amphotericin B deoxycholate with maximum tolerated dose being, 1 to 1.5 mg/kg/day. [10]. The nephrotoxic and acute infusional toxic effects of high dose conventional Amphotericin B frequently avoid long-term high-dose therapy. The optimal duration of therapy for mucormycosis remains poorly defined. Treatment decisions are highly customised. Most of the conventional azoles, including fluconazole and voriconazole, have no substantial activity against *Zygomycetes *fungi. Posaconazole, an orally existing wide-spectrum investigational triazole administered at a maximum dose of 800 mg/day in divided doses seems to possess potent antifungal activity [11]. Whether posaconazole alone or in synergism with a lyophilised formulation of Amphotericin B is favoured will require further research. The raised oxygen pressure achieved with HBO treatment seems to improve the capacity of neutrophils to kill organisms. In addition, by reversing lactic acidosis, treatment with HBO complements the oxidative action of Amphotericin B. HBO therapy for mucormycosis should comprise of exposure to 100% oxygen, each dive ranging from 90 minutes to 120 minutes at pressures from 2.0 to 2.5 atmospheres with 1 or 2 exposures on a daily basis for a total of 40 treatments [12]. Information on treatment of mucormycosis with HBO is scarce and its role is in doubt. The disease site and host factors are the primary determinants. There are no serological tests that can help with the diagnosis of mucormycosis. Negative biopsy samples and cultures, normalization of radiographic images of the affected site, and resurgence from immunosuppression are primary indicators that a patient is a candidate for stopping antifungal or adjunct forms of therapy.

## 4. Summary

Any individual with underlying immunocompromised status with suspected osteomyelitis of the jawbones should be investigated for fungal infections also. Correction of underlying predisposing factors and early diagnosis coupled with an aggressive multimodality treatment approach offer the best chance for survival in these patients. Aggressive surgical management in the form of ideal broad surgical debridement should be initiated early as most antifungal agents have poor penetration ability at the diseased site. 

## Abbreviation

HBO: Hyperbaric oxygen.
